# Competency-based medical education studying live anatomy by ultrasound

**DOI:** 10.5116/ijme.595f.b183

**Published:** 2017-07-19

**Authors:** Graziano Serrao, Massimo Tassoni, Alberto M. Magenta-Biasina, Antonio G. Mantero, Antonino M. Previtera, Michela C. Turci, Elia M. Biganzoli, Emanuela M. Bertolini

**Affiliations:** 1 Department of Health Sciences, San Paolo Medical School, Università degli Studi di Milano, Italy; 2 San Paolo Ultrasound Medical School (SPUMS), San Paolo Medical School, Università degli Studi di Milano, Milan, Italy; 3 ASST Santi Paolo e Carlo, Milan, Italy; 4 Department of Biomedical Sciences for Health, Università degli Studi di Milano, Milan, Italy; 5 Department of Clinical Sciences and Community Health, Università degli Studi di Milano, Milan, Italy

**Keywords:** Competency-based medical education, live anatomy, ultrasound, Italy

## Introduction

The evolution of healthcare demand poses serious problems to health services. It is the responsibility of the educators involved in the training of future physicians to find new strategies for the acquisition of adequate professional competences.

Nowadays, echography is the added value in many daily medical specialties, both in hospital and territorial contexts.

In order to implement a practical approach in the preclinical phase of the medical curriculum^1^^-^^7^, we practiced the study of anatomy in the living individual, defined as living anatomy, by inspection.^6^ Ultrasound was used to learn about the anatomy. As anatomists, we thought of this method as a virtual scalpel on a virtual dissection. It is safe, repeatable and devoid of degradation.

In undergraduate medical education, ultrasonography is an underemphasized diagnostic tool that is often limited to passive experiences. To compensate for this, we designed to begin as soon as possible, right from the first year of medical education.^1^ This educational project aims at forging the profile of a physician who can carry out first level echography. A century ago, physicians had to master stethoscopes. Graduates should now master miniaturized echoscopes.

### A shared educational project

A standardised curriculum which was developed by faculty experts in ultrasound was presented to the student body.^3^ Most students agreed to it. Since the academic year 2009, the San Paolo Medical School Didactic Council has approved vertically integrated courses of anatomy, technical skills training guided by peer tutors^8^^,^^9^ and ultrasound with an approved evaluation program.

From 2009 to 2016, all the six hundred and fifty-one students attending the first year were trained with this approach. Each year, 16 volunteers out of these students attended a preparatory course to become ‘Peer Tutors’ (PTs). Mentoring Doctors would then enable PTs to supervise ‘peer learners’ (PLs) autonomously. Pairs of PTs or PLs were alternately the subjects and the assessors of the study.

### Didactic plan

In the anatomy course, we adopted modules in which topography could favour the technology of the various probes from the surface (linear) to the deeper tissues (convex). Training included the study of the musculoskeletal system, major arterial and venous vessels, visceral spaces of the neck (thyroid, carotid arteries, jugular veins), most of the viscera contained in the chest (heart), abdomen (liver, gallbladder, spleen, pancreas, kidney, aorta and branches, inferior vena cava and its tributaries, portal vein and its tributaries), and pelvis (bladder, uterus, ovary).^10^ The course was supplied as follows:

1) Palpatory living anatomy: our intention was not to compete with clinical colleagues in teaching semeiotics. Indeed, we would improve the exploration of the body forms by all disposable means along the cognitive path of anatomy. Each student dedicated 50 hours to attend this module: 20h were assisted by a tutor and 30h were self-study and self-training. 2) Ultrasound scanning knobology. 3) Musculoskeletal system, major neurovascular Bundles, thyroid gland (Ms/nvB) echography survey by means of a linear multi-frequency probe equipped ultrasound machine (Logic QE GE Medical Systems, Milwaukee, WI, USA).

During dedicated probe-in-the-hand sessions, each student practiced controlling the correspondence between palpatory detection and target structures (e.g.: rotator cuff tendons, ulnar nerve in the elbow, median nerve in the wrist, neurovascular bundles). Students were taught to recognize the deeper structures of clinical interest which are hardly traceable by palpation (e.g., deep flexors/extensors of forearm/leg; carpal tunnel). Forty hours were devoted to this module (20h tutored; 20h self-training). 4) Thoracic, abdominal and pelvic viscera. Students were taught to scan correct and complete images from the organ of interest. For instance, they had to perform the following ultrasound projections of the heart: parasternal long axis and parasternal short axis at the level of the semilunar cusps of the aortic valve, at the level of the mitral cusps and at the level of the papillary muscles; the apical four chambers and the two chambers.^11^ For liver scanning, we followed a sub-costal epigastric approach to study the left lobe and a transverse sub-costal or inter-costal approach to scan the right lobe. To explore and identify sectors, the liver was scanned with optimal windows to obtain the confluence of hepatic veins on screen. Liver segments were studied by observing right and left branches of the portal vein.^12^ Spleen and kidneys were explored through long and short axes. Renal vascular segments were also studied. Positions, courses and main branches of major abdominal vessels were then explored. This module required 80h (40h with PTs, 40h self-study and self-training). Students practiced the survey using a pocket-sized ultrasound machine (VScan GE) as well as a Logic QE. Competences were verified by experienced sonographers.

### What is the lesson?

From our experience, the correspondence between anatomy palpation and ultrasound windows allowed optimal skills. Conversely, the studies on deep structures were more challenging. Ultrasonography applied to virtual dissection had the undoubted advantage of focusing on one part of the body, as often happens in clinical practice, while observing the structures of interest from an optimal window. Autonomous exercise allowed the students to make their mistakes, compare results and enhance their own skills. This approach improved self-criticism and focused the attention on organ shapes to achieve well standardised quality. The students considered the teaching of anatomy by ultrasound an innovative, exciting and engaging approach. Through the implementation of peer education, the project has proved to be a model which could be used by all the students of the course.

## Conclusions

Over the seven years and throughout the different modules, the level of expertise reached by PTs and PLs was generally satisfactory.

Evidence-based echography applications in care practices have increased. It is time to strive for the integration of ultrasonography into the medical school curricula. Moved by evidence, teachers must work hard to transform excellence into routine performances, to warranty assistance on site and to enhance quality in the spending review regimen. However, the main objective when training future physicians to handle ultrasonography, as well as preparing them for their professional life with the necessary expertise in using personal stethoechoscopes, was successfully started.^13^

### Acknowledgements

The authors would like to acknowledge the mother tongue Ms Sara W. Grassi, experienced in scientific academic English, for her invaluable support in the complete and accurate revision of the manuscript.

### Conflict of Interest

The authors declare that they have no conflict of interest.
